# Combined Oral Contraceptive Effects on Low-Grade Chronic Inflammatory Mediators in Women with Polycystic Ovary Syndrome: A Systematic Review and Meta-Analysis

**DOI:** 10.1155/2018/9591509

**Published:** 2018-11-25

**Authors:** Sebastião Freitas de Medeiros, Matheus Antônio Souto de Medeiros, Nayara de Souza Santos, Bruna Barcelo Barbosa, Márcia Marly Winck Yamamoto

**Affiliations:** ^1^Department of Gynecology and Obstetrics, Medical School, Federal University of Mato Grosso, Cuiabá, MT, Brazil; ^2^Tropical Institute of Reproductive Medicine, Cuiabá, MT, Brazil

## Abstract

Polycystic ovary syndrome is associated with dyslipidemia, dysglycemia, metabolic syndrome, and low-grade chronic inflammation, which increase the risks for cardiovascular disease. Combined oral contraceptives may affect the mediators of low-grade chronic inflammation with potential additive risk in PCOS patients. This meta-analysis investigates the impact of oral contraceptive on markers of chronic inflammation in PCOS patients. Pubmed, Scopus, and Cochrane database were used to search studies reporting on this matter in the target population. Twenty seven studies were selected, including a total of 838 women. The data were expressed as the standardized mean difference. The random-effects model was used to summarize effect sizes. Heterogeneity was examined using Cochran's test (Q) and I^2^ statistics. Most of the preparations increased C-reactive protein (CRP) in PCOS patients (p >0.001). The increase in homocysteine levels was not significant (p >0.05). Follistatin significantly increased with pills containing cyproterone acetate (p= 0.008). Interleukin-6 changes were inconsistent and plasminogen activator inhibitor-1 decreased with pills containing desogestrel, norgestimate, and drospirenone. Collectively, the results of this review indicate that oral contraceptives modify most inflammatory markers of PCOS patients. However, the clinical implications are not clear yet and future studies must consider longer follow-up and the inclusion of objective clinical parameters.

## 1. Introduction

Polycystic ovary syndrome (PCOS), which is common in women of reproductive age, presents with different phenotypes regarding androgen levels, oligo-/anovulation, ovary morphology, dyslipidemia, dysglycemia, and fat body mass. Basically, four phenotypes are clearly defined: hyperandrogenism, oligo- or anovulation plus polycystic ovary morphology (A), hyperandrogenism plus oligo-/anovulation (B), oligo-/anovulation plus polycystic ovary morphology (C), and hyperandrogenism plus polycystic ovary morphology (D). Clinical and/or biochemical hyperandrogenism, abnormal fat distribution, central obesity, and insulin resistance are observed in 50%-70% and metabolic syndrome in about 40% of PCOS patients [1]. The different phenotypes of PCOS may induce endothelial dysfunction and atherosclerosis [2]. Low-grade chronic inflammation is characterized by increased blood levels of several mediators of endothelial dysfunction and inflammatory processes. The principal markers of the inflammatory state include C-reactive protein (CRP), tumor necrosis factor alpha (TNF-*α*), homocysteine (Hcy), interleukins 1 and 6 (IL-1, IL-6), follistatin, and total number of white blood cells (WBCs) [3–8]. The initiation of the inflammatory process is complex but it is tightly associated with body mass and android fat distribution [1, 9].

Despite the complex pathophysiology and multiple phenotypes of PCOS, the first-line treatment of all women with PCOS not planning to become pregnant is combined oral contraceptives (COCs). The major risks and benefits of COCs in PCOS patients were recently reviewed [10]. Because COC use itself may induce an inflammatory profile [11], the combination of oral contraceptives and PCOS could worsen or increase the risks for venous thromboembolism and cardiovascular disease (CVD) in these patients. The main objective of this meta-analysis was to examine the potential additional risk of all COC preparations in women with PCOS by investigating each markers of low-grade chronic inflammation.

## 2. Methods and Methods

### 2.1. Search Procedure

To answer the question whether combined oral contraceptives modify or not inflammatory markers, a search and review of all studies published between 1990 and 2017 on the impact of COC on inflammatory markers in PCOS patients that were written in English, using PubMed, Scopus, and the Cochrane database were done. Keywords in different combination for the search included oral contraceptive, polycystic ovary syndrome, polycystic ovary disease, polycystic ovarian disease, chronic inflammation, inflammatory mediators, and contraception. Online searches of specialized journals were also used by adding appropriate keywords. In addition, these databases were expanded by manual search of reference lists of relevant studies from the obtained articles.

### 2.2. Criteria for Study Inclusion

Study inclusion was based on a description of the PCOS diagnostic using the National Institute of Health (NIH) or the Rotterdam criteria and comparison of the inflammatory markers concentrations before and after use of any COC formulation for at least three months. Eligibility was double-checked independently by two authors (SFM, MMWY). Taking into account the included studies, the effects of COCs on inflammatory mediators were evaluated in 838 PCOS women by comparison of the data obtained before and after 3-18 months of COC use.

### 2.3. Data Extraction and Quality Assessment

Two authors (SFM, MMWY) extracted information regarding publication intervention, study design, outcome, statistical methods, and results. Further, they independently assessed quality and bias using the Newcastle-Ottawa scale for evaluating the quality of randomized and nonrandomized observational studies in meta-analysis [12]. As recommended three factors were considered to score the quality of included studies: (A) selection (representativeness of exposed cohort, selection of no exposed cohort, and ascertainment of exposure), (B) demonstration (at the start of the study the outcome of interest of modified), comparability (assessed on the bias of study design and analysis), and (C) outcome (assessment of outcome, sufficient follow-up, and adequate follow-up). The quality of the studies was rated good (3 stars in  a, to 2 stars in  b, and 2 to 3 stars in  c), fair (2 stars in  a; 1 to 2 stars in  b, and 2 to 3 stars in  c), and poor (0 to 2 stars in  a, 0 stars in  b, and 0 to 1 stars in  c) (Table 1).

### 2.4. Outcome Measures

As the primary outcomes were changes in the concentrations of CRP, Hcy, follistatin, TNF-*α*, IL-6, leukocyte total number, and several adhesion molecules with the use of COCs, only studies in which individuals were used as their own control were included.

### 2.5. Data Analysis

Because studies did not use the same assays to measure the principal marker (CRP), the effect sizes are presented as the standardized mean difference (SMD) and standard error (SE). When adequate these methods were used for analysis of the inflammatory markers. Data were pooled by using a random-effects model. Under this model, it was assumed that the true effect size may vary from study to study, and the summary effect was the estimate of the mean of the distribution of effect sizes [13]. The variance of the effect size across the studies (Tau^2^) was estimated using the method of moments [14]. Heterogeneity was assessed for each result by using Cochran's Q statistic and the I^2^ score to quantify the degree of heterogeneity [15]. When pooling the results of different studies was not possible, the analysis of the effect size in a particular study was described as the raw mean difference reported in the original studies, and these differences were analyzed using ANOVA, two-sided Student's t tests, or Wilcoxon or Mann-Whitney tests as described in each study.

## 3. Results

### 3.1. Studies Selection

**Figure 1:**
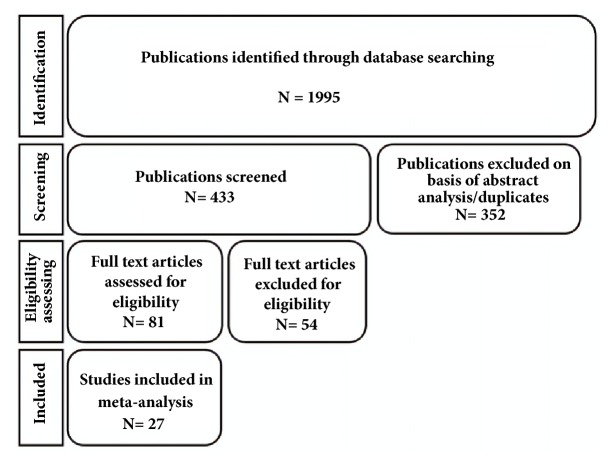
Flow chart.

A total of 1995 articles were initially identified. After screening by one of the authors (SFM) using the reported keywords, 433 studies were maintained. Three reviewers (NSS, MMWY, and MASM) screened abstracts to assess the eligibility of the studies. With this procedure, 1562 articles were excluded (Figure 1). Furthermore, another 54 full articles were excluded because they did not compare the values of the variables of interest obtained before and after the use of COCs or did not provide sufficient data for analysis. Finally, 27 full observational papers providing all necessary data were selected (Table 2).

### 3.2. Risk of Bias

The study quality was verified using the risk of bias assessment tool Newcastle-Ottawa scale for observational studies and summarized in Table 2.

### 3.3. Effects of Interventions

The pooled results of the studies are presented in the following sections. In some sections, the results were not pooled and instead are presented as single mean differences. Tables 3 and 4 summarize the effects of interventions using different COC formulations.

#### 3.3.1. C- Reactive Protein

**Figure 2:**
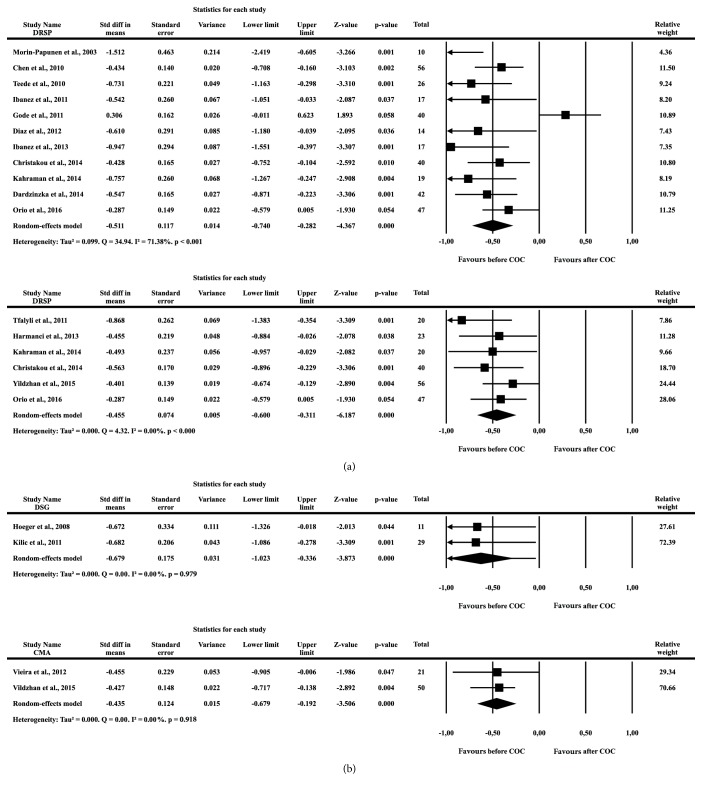
(a) Forest plot of the standardized difference means of C-reactive protein taken before and after the use of combined oral contraceptive in PCOS patients. (b) Forest plot of the standardized difference means of C-reactive protein taken before and after the use of combined oral contraceptive in PCOS patients.

Data regarding the impact of COCs containing 35 *μ*g ethinylestradiol (EE) and 2 mg cyproterone acetate (CPA) on CRP levels of PCOS patients after 3-6 months of use were extracted from eleven articles, which were published in peer-reviewed journals between 2003 and 2016 [5, 16–25]. All studies were randomized or nonrandomized open trials. A total of 328 PCOS patients were enrolled, and their CRP levels were compared before and after the COC use. The SMD was -0.511 (SE = 0.117, p <0.001) (Figure 2(a)). Perhaps because different assays were used to measure CRP (standard or highly sensitive), these studies were heterogeneous (Tau^2^ = 0.099, Q = 34.94, I2 = 71.38, p <0.001).

Changes in the serum concentrations of CRP before and after the use of a combined formulation containing 30 *μ*g EE and 3 mg drospirenone (DRSP) for 3-6 months were reported in six studies published between 2011 and 2016 [22, 23, 25–28]. A total of 206 PCOS patients were enrolled, and the SMD was -0.455 (SE=0.074, p <0.001). These studies were homogeneous (Tau^2^ = 0.000, Q = 4.32, I^2^ =0.00, p <0.001) (Figure 2(a)). Comparison of the difference in CRP levels before and after the use of a combination containing 30 *μ*g EE and 2 mg CMA was performed in two reports that included 71 PCOS patients [28, 29]. Either a significant increase of 195% [29] or a nonsignificant increase of 12% was reported [28]. The SMD was -0.435 (SE = 0.124, p <0.001). Both studies were highly homogeneous (Tau^2^ = 0.00, Q = 0.01, I^2^ = 0.00, p = 0.918) (Figure 2(b)).

The impact of 150 *μ*g of the progestin desogestrel (DSG) combined with 30 *μ*g EE on CRP levels was verified in two studies published in 2008 and 2011, which included only 60 PCOS patients [30, 31]. The SMD was -0.059 (SE = 0.429, p = 0.891). The studies were highly heterogeneous (Tau^2^ = 0.486, Q = 17.52, I^2^ = 88.58, p <0.001) (Figure 2(b)). A combination of 20 *μ*g EE and 150 *μ*g DSG for 3 months induced a nonsignificant increase in the CRP level of PCOS patients from 1.6 mg/l to 1.70 mg/l (p = 0.720) [32]. A single study [33] reported a nonsignificant increase in CRP levels from 4.9 mg/l to 5.6 mg/l (p >0.05) after the use of a combination of 30 *μ*g EE and 0.15-0.25 mg of the progestin norgestimate (NGM).

#### 3.3.2. Homocysteine

**Figure 3:**
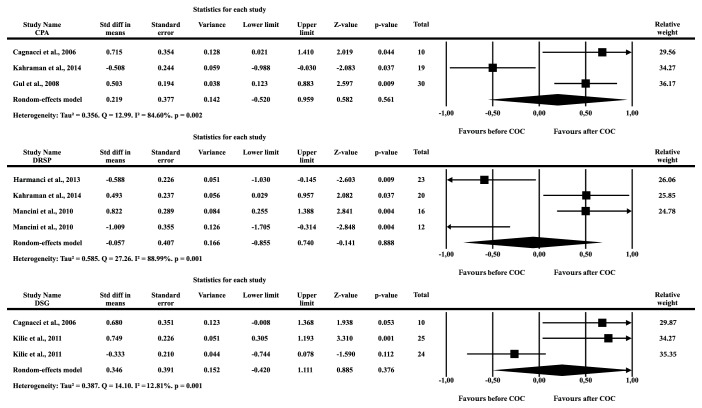
Forest plot of the standardized difference means of homocysteine between before and after the use of combined oral contraceptive in PCOS patients.

Three studies with PCOS patients using a combination of 35 *μ*g EE and 2 mg CPA reported increases in Hcy levels of 6%-18% [23, 34, 35]. Comparison of the Hcy levels before and after the COC administration performed in these studies, with 59 PCOS patients, showed SMD of 0.219 (SE = 0.377, p = 0.561). The studies were highly heterogeneous (Tau^2^ = 0.356, Q = 12.99, I^2^ = 4.60, p = 0.002) (Figure 3).

COCs with DRSP were reported to either decrease or increase Hcy levels [23, 27, 36]. The analysis combining the results of 71 PCOS patients demonstrated an SMD of -0.057 (SE = 0.407, p = 0.888), and the studies were highly heterogeneous (Tau^2^ = 0.585, Q = 27.26, I^2^ = 88.89, p = 0.001) (Figure 3). The combination of 30 *μ*g EE and 150 *μ*g DSG decreased or did not change the Hcy levels in PCOS users in two studies [31, 34]. When obese and nonobese PCOS patients were taken into account, the SMD was 0.346 (SE = 0.391, p = 0.376). These studies were also shown to be highly heterogeneous (Tau^2^ = 0.387, Q = 14.10, I^2^ = 85.81, p = 0.001) (Figure 3).

#### 3.3.3. Follistatin

**Figure 4:**
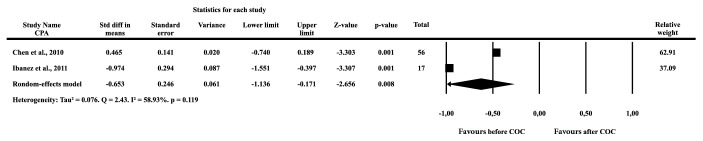
Forest plot of the standardized difference means of follistatin before and after the use of combined oral contraceptive in PCOS patients.

Two studies examined follistatin changes after the use of COCs containing 35 *μ*g EE and 2 mg CPA in 73 PCOS patients [5, 18] and, in both studies, the follistatin levels were significantly increased (p <0.001 for both). The SMD in follistatin levels was -0.653 (SE = 0.246, p = 0.008). The studies only showed a moderate nonsignificant heterogeneity (Tau^2^ = 0.076, Q = 2.43, I^2^ = 58.93, p = 0.119) (Figure 4).

#### 3.3.4. Interleukin-6

Five studies with 84 PCOS patients examined changes in IL-6 with the use of different COC compositions [20, 29, 33, 37, 38] but it was not possible to pool the data, and the effect sizes are reported individually as single mean differences (Table 4). A nonsignificant decrease in IL-6 levels, from 1.67 pg/ml to 1.39 pg/ml (ES = -0.280; p = 0.911), was reported with the use of 35 *μ*g EE and 2 mg CPA [20]. PCOS patients using COCs with DRSP showed a nonsignificant increase in IL-6 levels, from 0.95 pg/ml to 1.09 pg/ml (ES = 0.140; p >0.05), after nine months of use [37]. One study found a nonsignificant decrease in IL-6 levels in users of COCs containing CMA, from 1.9 pg/ml to 1.7 pg/ml (ES = -0.200; p >0.05) [29]. After the use of COCs containing the progestin DSG for 12 months, IL-6 levels showed a nonsignificant decrease from 1.7 pg/ml to 1.4 pg/ml (ES= -0.300; p >0.05). Use of COCs with NGM for three months by PCOS patients resulted in a nonsignificant decrease in IL-6 levels from 2.1 pg/ml to 1.6 pg/ml (ES = -0.500; p = 0.060). Collectively, COCs do not increase the levels of IL-6 in PCOS patients. The results of the studies are limited because the women used different COC compositions and studies had a small number of patients.

#### 3.3.5. Plasminogen Activator Inhibitor-1

The use of a combination of 30 *μ*g EE and 150 *μ*g DSG resulted in a significant decrease in the PAI-1 levels after six months of use from 46.9 ng/ml to 29.50 ng/ml (ES = -17.4; p <0.05) [30]. A pill containing 35 *μ*g EE and 0.18-0.25 *μ*g NGM induced a nonsignificant decrease in PAI-1 levels, from 81.2 ng/ml to 76.5 ng/ml (ES = -4.7; p = 0.520), after three months of use by PCOS patients [33]. Furthermore, a combination of 30 *μ*g EE and 3 mg DRSP also resulted in a nonsignificant decrease in PAI-1 levels from 2.6 UI/ml to 2.4 IU/ml (ES = -0.20; p >0.05) after six months of use [25]. Collectively, COCs decrease PAI-1 levels in PCOS patients, but studies with larger sample size are still needed to definitively establish the clinical significance of this change. In another study, which was not included in this meta-analysis due to missing data, PAI-1 levels were significantly decreased after six months of use of 35 *μ*g EE and 2 mg CPA by PCOS patients (ES = -0.7 IU/ml, p = 0.004) [17].

#### 3.3.6. Leukocyte Total Number

In a randomized open clinical trial, COC preparations with 30 *μ*g EE and 3 mg DRSP or 75 *μ*g GSD (pooled) demonstrated a nonsignificant increase in the total neutrophil count, from 3.9 x 1000/mm^3^ to 4.7 x 1000/mm^3^ (ES = 0.80; p >0.05) and a nonsignificant decrease in lymphocytes from 2.3 x 1000/mm^3^ to 2.2 x 1000 mm^3^ (ES = -0.10; p >0.05) [3] (Table 2). Therefore, COCs might not increase leukocyte number in PCOS users.

#### 3.3.7. Tumor Necrosis Factor-*α*

Only a single study reported the impact of COCs on TNF-*α* in PCOS subjects. A combination of 30 *μ*g EE and 2 mg CMA showed a nonsignificant increase of 10.6 pg/ml to 12.0 pg/ml (ES = 1.4; p >0.05) in TNF-*α* levels in PCOS patients [29] (Table 4).

#### 3.3.8. Other Chronic Inflammatory Markers

The influence of COCs on MCP-1 was evaluated in a single study [38] and this study, using a combination of 30 *μ*g EE and 150 *μ*g DSG, showed a nonsignificant decrease of MCP-1 from 50 pg/ml to 44 pg/ml (ES = -0.60; p >0.05). The impact of a combination of 35 EE *μ*g and 2 mg CPA for three months on several adhesion molecules markers in PCOS women was also described in a single study [39]. With the EE/CPA combination, soluble intracellular adhesion molecule-1 (sICAM-1) concentrations decreased from 370.1 ng/ml to 364.7 ng/ml (ES = -5.4; p = 0.800), and vascular cell adhesion molecule-1 (sVCAm-1) decreased from 776.3 ng/ml to 742.9 ng/ml (ES = -33.4; p = 0.100) with EE/CMA [32]. A combination of 20 *μ*g EE and 150 *μ*g DSG used for three months significantly decreased sVCAM-1 in PCOS patients, from 583 ng/ml to 522 mg/l (ES = -61.0; p = 0.003) [39]. With EE/CPA, se-selectin increased from 29.1 ng/ml to 37.1 ng/ml (ES = 8.0; p = 0.100), and sp-selectin decreased from 229.4 ng/ml to 189.6 ng/ml (ES = -39.8; p = 0.080). Thus, COCs did not negatively influence the levels of adhesion molecules in PCOS users and could even improve their levels in these patients. COCs containing 35 *μ*g EE and 2 mg CPA were shown to significantly increase sCD40L from 1.33 ng/ml to 2.7 ng/ml (ES = 1.37; p = 0.011) after three months of use in PCOS subjects [40]. Table 2 summarizes all the changes in markers of chronic inflammation following the use of different COCs in PCOS subjects.

## 4. Discussion

Given the frequent prescription of COCs as first-line treatment for PCOS women not seeking to become pregnant and the possible influence of COCs on low-grade chronic inflammatory markers in these patients, this meta-analysis was justified. The present study examined the data from a total of 27 reports with the inclusion of over than eight hundred PCOS women. Despite the design including patients as their own control, most reports suffer from small sample size with the inclusion of less 50 patients. Unfortunately, some data could not be pooled, and the effect sizes were given individually, according to the different COC compositions. The meta-analysis showed that most COCs were associated with worsening CRP concentrations, nonsignificant changes in Hcy levels, increase in follistatin, and conflicting results regarding IL-6 concentrations. It was also found that COCs did not change significantly the leukocyte total number and TNF-*α* levels. Otherwise, COCs tended to decrease PAI-1 and adhesion molecule levels in the blood.

The small sample sizes for comparisons of some inflammatory mediators must be considered. The short-term follow-up times, which were frequently limited to three months, must also be taken into account before definitive conclusions. Finally, the heterogeneity between studies indicates that the results must be interpreted with caution. The strengths of the current study include the evaluation of several mediators of chronic inflammation, the use of established criteria for PCOS diagnosis, and the medium-to-high quality requirement for inclusion of studies. To overcome the impact of the heterogeneity due to clinical or methodological issues, the SMD and a random-effects model were used to compensate for heterogeneity among the studies.

CRP was reported to be 2-fold higher in PCOS patients than controls and is considered a major predictor of metabolic dysfunction and low-grade chronic inflammation in PCOS subjects [41]. Moreover, an additional increase in CRP levels with the use of COCs was reported in these patients [7] and this increase in CRP levels is believed to be due to a direct effect from increased hepatocyte synthesis rather than from the IL-6-dependent inflammatory process [5, 7, 24, 42]. The results of the current meta-analysis endorse previous findings and demonstrate that CRP is increased with the use of COCs. Among eleven studies reporting on CRP levels in PCOS users of COCs containing EE/CPA, only one did not report an increase in this protein [42].

PCOS users of COCs containing EE/DRSP have also been reported to have increased CRP levels [23, 25, 27]. The current meta-analysis has confirmed a significant increase in CRP with this formulation. One single comparison showed that the changes in CRP levels were higher with EE/CMA than the increase observed with the EE/DRSP combination [28]. This meta-analysis also demonstrated that pills containing EE/DSG resulted in a nonsignificant increase in CRP in either lean or obese PCOS patients [30, 31].

Overall, the results of this review demonstrate that the use of COCs by PCOS women increases CRP independent of the formulation and could be associated with an increased risk of atherogenesis. In addition, these findings suggest that the CRP increase varies with the progestin or the estrogen dose [43]. In summary, COCs maintain or even worsen the CRP levels in PCOS women. However, it is yet unclear if the elevated CRP levels observed after COC therapy in women with PCOS actually represent aggravation of the inflammatory process in target tissues [5]. A positive correlation among CRP concentrations, endothelial dysfunction, and the severity of the atherosclerotic process have been reported in several studies [7, 42].

Independent of other risk factors, hyperhomocysteinemia is a recognized risk marker for atherosclerosis [44] due to its capacity to increase oxidative stress in the vascular endothelium and activate platelets [45]. Hcy has been reported to be increased in PCOS patients [46]. The effects of different COC formulations for 3-12 months on Hcy levels in PCOS patients are inconsistent and it seems to depend on the specific composition. The studies with COCs containing EE and CPA have been reported to either increase [23] or decrease [34, 35] Hcy concentrations in PCOS users. In the current meta-analysis, a nonsignificant reduction in Hcy levels was demonstrated with pills containing CPA. COCs containing the progestin DSG also resulted in a small nonsignificant decrease in Hcy levels [31, 34]. Oral contraceptives containing the progestin DRSP did not significantly modify the Hcy levels in PCOS subjects. Regarding Hcy, the current data showed that COCs tend to decrease Hcy levels of PCOS subjects.

Acting as an antagonist of aromatase activity, the glycosylated polypeptide follistatin has been reported to facilitate PCOS via a polymorphism in its gene [47]. Follistatin levels were found to be increased in PCOS women, either obese and nonobese [5, 48]. The increased levels of follistatin in PCOS users of COCs may be attributed to increased secretion from hepatocytes rather than to an acute inflammatory response. However, the impact of different COCs on follistatin concentrations in PCOS patients has rarely been reported. A combination of EE and CPA resulted in a significant increase in follistatin in PCOS patients [5, 18]. In young hyperandrogenemic and hyperinsulinemic patients who were not diagnosed with PCOS, follistatin was also reported to be increased by nearly 4-fold after treatment with COCs containing EE and DRSP [18]. The current meta-analysis confirmed a significant increase in follistatin levels of PCOS users of COCs containing the progestin CPA. COCs containing other progestins have not been examined in PCOS women. Therefore, more studies including clinical variables are needed to evaluate the impact of follistatin changes on the cardiovascular system of PCOS women.

The increased IL-6 levels previously observed in PCOS seem to be related to obesity and not to PCOS itself [41]. A nonsignificant decrease in IL-6 of PCOS women has been observed after using different COC formulations [20, 29, 33, 38]. Only one study reported a small and nonsignificant increase in IL-6 with the use of COCs containing the progestin DRSP [37]. Thus, it can be concluded that COCs do not significantly change IL-6 concentrations in PCOS women.

The increased number of leukocytes, primarily neutrophils, observed in PCOS is likely a result of hyperandrogenism [3, 49]. The relative increase in neutrophils is believed to be amplified by COC use. In the current review, the use of a COC formulation containing EE and DRSP did not result in significant changes in total number of neutrophils and lymphocytes [3]. So, regarding the use of COCs by PCOS patients and leukocyte number, no conclusion can be made at this time, but it is likely that COCs do not modify leukocytes in this condition.

TNF-*α* participates in the pathogenesis of insulin resistance and is elevated in both obese and nonobese PCOS women [50]. A COC preparation with 30 *μ*g EE and 2 mg CMA nonsignificantly increased TNF-*α* in PCOS users [29]. Although TNF-*α* is considered an important marker of low-grade chronic inflammation, more studies are needed regarding the use of COC in PCOS, and, due to the paucity of data, no conclusion can be drawn at this time. Several adhesion molecules may be higher in PCOS patients than in healthy subjects [51] and could be a risk factor for atherosclerosis and chronic inflammation in these patients. The influence of COCs on the concentrations of these molecules in PCOS women was reported in a few studies [32, 38]. Only a combination of 30 *μ*g EE and 0.15 mg DSG resulted in a significant decrease in sVCAM-1 levels in PCOS users [38]. Moreover, COCs containing the progestins CPA and CMA did not significantly change sICAM-1, sVICAm-1, and selectins.

PAI-1 levels have been reported to decrease with the use of COCs containing different progestins. Specifically, this decrease was significant with the progestins CPA and DSG [30]. Though, in the current review, the raw effect size varied from -0.2 [25] to -17.4 UI/ml [30], the data could not be pooled for analysis. The extent to which decrease in PAI-1 may improve endothelial function in PCOS patients is still unclear and may be mediated by diminishing its prothrombotic, antithrombotic, and vascular smooth muscle proliferation effects. The weak negative effect of COCs on insulin resistance does not negate the estrogen-induced beneficial effect on PAI-1 [17].

## 5. Conclusion

In summary, the current study demonstrated that the studies included in the meta-analysis have fair to good quality, although the follow-up had been limited to 3-18 months. C-reactive protein concentrations increased with most preparations of combined oral contraceptives, mainly with the most frequently used in PCOS patients. The decrease in plasminogen activator inhibitor-1 levels with COCs containing DSG, NGM, and DRSP must be evaluated regarding the clinical relevance and impact on the cardiovascular system. Finally, it was demonstrated that most inflammatory markers are modulated by COCs use in PCOS women but the clinical implication for practice needs more extensive investigation and future studies should consider the inclusion of larger sample size, much longer follow-up periods, and the inclusion of objective clinical parameters in their outcome.

## Figures and Tables

**Table 1:** Characteristics of clinical studies comparing before and after changes in markers of chronic inflammation in PCOS patients with the use of different combined oral contraceptives.

Study	Country	Study design	N°	COC	Duration	PCOS
			patients	type and dose	months	diagnostic criteria
Morin-Papunen et al, 2003	Finland	Randomized open trial	10	EE 35 *μ*g/ CPA 2 mg	06	NIH^a^
Ibanez and de Zegher, 2004	Belgium/ Spain	Randomized open trial	16	EE 30 *μ*g/ DRSP 3 mg	09	Rotterdam^a^
Ibanez et al, 2005	Spain/ Belgium	Randomized open trial	21	EE 30 *μ*g/ DRSP 3 mg	03	Rotterdam^a^
Cagnacci et al, 2006	Italy	Randomized open trial	10	EE 35 *μ*g/ CPA 2 mg	06	NIH^a^
Cagnacci et al, 2006	Italy	Randomized open trial	10	EE 30 *μ*g/ DSG 0.15 mg	06	NIH^a^
Banaszewska et al, 2007	USA	Crossover open trial	24	EE 20 *μ*g/ DSG 0.15 mg	03	Rotterdam
Gul et al, 2008	Turkey	Non-randomized open trial	30	EE 35 *μ*g/ CPA 2 mg	03	Rotterdam
Hoeger et al, 2008	USA	Randomized open trial	11	EE 30 *μ*g/ DSG 0.15 mg	06	NIH^a^
Bilgir et al, 2009	Turkey	Randomized open trial	20	EE 35 *μ*g/ CPA 2 mg	03	Rotterdam
Kebapcilar et al, 2010	Turkey	Randomized open trial	28	EE 35 *μ*g/ CPA 2 mg	03	Rotterdam
Chen et al, 2010	Taiwan	Non-randomized open trial	56	EE 35 *μ*g/ CPA 2 mg	03	Rotterdam
Mancini et al, 2010	Italy	Non-randomized open trial	28	EE 30*μ*g/ DRSP 3 mg	06	Rotterdam
Teede et al, 2010	Australia	Randomized open trial	26	EE 35 *μ*g/ CPA 2 mg	06	NIH
Tfayli et al, 2011	USA	Non-randomized open trial	20	EE 30 *μ*g/ DRSP 3 mg	06	NIH
Essah et al, 2011	USA	Randomized open trial	10	EE 35 *μ*g/ NGM 0.18-0.25 mg	03	Rotterdam^a^
Kilic et al, 2011	Turkey	Randomized open trial	49	EE 30 *μ*g/ DSG 0.15 mg	06	Rotterdam
Ibanez et al, 2011	Belgium/ Spain	Randomized open trial	17	EE 35 *μ*g/ CPA 2 mg	06	NIH^a^
Gode et al, 2011	Turkey	Non-randomized open trial	40	EE 35 *μ*g/ CPA 2 mg	06	Rotterdam
Vieira et al, 2012	Brazil	Randomized open trial	21	EE 30 *μ*g/ CMA 2 mg	12	Rotterdam
Diaz et al, 2012	Spain/ Belgium	Randomized open trial	14	EE 35 *μ*g/ CPA 2 mg	12	NIH^a^
Harmanci et al, 2013	Turkey	Non-randomized open trial	23	EE 30 *μ*g/ DRSP 3 mg	06	Rotterdam
Ibanez et al, 2013	Spain/ Belgium	Randomized open trial	17	EE 35 *μ*g/ CPA 2 mg	18	NIH^a^
Christakou et al, 2014	Greece	Non-randomized open trial	40	EE 35 *μ*g/ CPA 2 mg	06	NIH
Christakou et al, 2014	Greece	Non-randomized open trial	40	EE 30 *μ*g/ DRSP 3 mg	06	NIH
Glintborg et al, 2014	Denmark	Randomized open trial	23	EE 30 *μ*g/ DSG 0.15 mg	12	Rotterdam
Kahraman et al, 2014	Turkey	Randomized open trial	19	EE 35 *μ*g/ CPA 2 mg	12	AES
Kahraman et al, 2014	Turkey	Randomized open trial	20	EE 30 *μ*g/ DRSP 3 mg	12	Rotterdam
Dardzinska et al, 2014	Finland	Crossover open trial	42	EE 35 *μ*g/ CPA 2 mg	04	Rotterdam
Yildzhan et al, 2015	Turkey	Randomized open trial	56	EE 30 *μ*g/ DRSP 3 mg	06	Rotterdam
Yildzhan et al, 2015	Turkey	Randomized open trial	50	EE 30 *μ*g/ CMA 2 mg	06	Rotterdam
Orio et al, 2016	Italy	Randomized double-blind trial	47	EE 35 *μ*g/ CPA 2 mg	06	Rotterdam^a^

a: not clearly stated.

**Table 2:** Risk of bias assessment of the included studies^a^.

**Study, Year**	**Selection**				**Comparability of cohorts (matched for)**	**Outcome**			**Total score**
	**Representativeness of exposed cohort**	**Selection of nonexposed cohort**	**Ascertainment of exposure**	**Outcome not present at baseline**		**Assessment of outcome**	**Sufficient follow-up duration**	**Adequate follow-up**	
Morin-Papunen et al, 2003	∗	∗	∗	–	BMI, age^∗∗^	∗	∗	∗	8
Ibanez and de Zegher, 2004	∗	∗	∗	–	BMI^∗^	∗	∗	∗	7
Ibanez et al, 2005	∗	∗	∗	–	BMI, age^∗∗^	∗	–	∗	7
Cagnacci et al, 2006	∗	∗	∗	–	BMI, age^∗∗^	∗	∗	∗	8
Banaszewska et al, 2007	∗	∗	∗	–	Age^∗^	∗	–	∗	6
Gul et al, 2008	∗	∗	∗	–	BMI, age^∗∗^	∗	–	∗	7
Hoeger et al, 2008	∗	∗	∗	–	BMI, age^∗∗^	∗	∗	∗	8
Bilgir et al, 2009	∗	∗	∗	–	BMI, age^∗∗^	∗	–	∗	7
Kebapcilar et al, 2010	∗	∗	∗	–	BMI, age^∗∗^	∗	–	∗	7
Chen et al, 2010	∗	∗	∗	–	Weight, BMI, age^∗∗∗^	∗	–	∗	8
Mancini et al, 2010	∗	∗	∗	–	BMI^∗^	∗	∗	∗	7
Teede et al, 2010	∗	∗	∗	–	BMI, age^∗∗^	∗	∗	∗	8
Tfayli et al, 2011	∗	∗	∗	–	BMI, age, fat mass^∗∗∗^	∗	∗	∗	9
Essah et al, 2011	∗	∗	∗	–	BMI^∗^	∗	–	∗	6
Kilic et al, 2011	∗	∗	∗	–	BMI^∗^	∗	∗	∗	7
Ibanez et al, 2011	∗	∗	∗	–	BMI, age^∗∗^	∗	∗	∗	8
Gode et al, 2011	∗	∗	∗	–	BMI, WC, age^∗∗∗^	∗	∗	∗	9
Vieira et al, 2012	∗	∗	∗	–	BMI, age^∗∗^	∗	∗	∗	8
Diaz et al, 2012	∗	∗	∗	–	BMI, age^∗∗^	∗	∗	∗	8
Harmanci et al, 2013	∗	∗	∗	–	BMI, WHR, age^∗∗∗^	∗	∗	∗	9
Ibanez et al, 2013	∗	∗	∗	–	BMI, age^∗∗^	∗	∗	∗	8
Christakou et al, 2014	∗	∗	∗	–	BMI, age^∗∗^	∗	∗	∗	8
Glintborg et al, 2014	∗	∗	∗	–	BMI, fat mass^∗∗^	∗	∗	∗	8
Kahraman et al, 2014	∗	∗	∗	–	BMI, WHR, age^∗∗∗^	∗	∗	∗	9
Dardzinska et al, 2014	∗	∗	∗	–	BMI, age^∗∗^	∗	∗	∗	7
Yildzhan et al, 2015	∗	∗	∗	–	BMI, WHR, age^∗∗∗^	∗	∗	∗	9
Orio et al, 2016	∗	∗	∗	–	BP, BMI, age^∗∗∗^	∗	∗	∗	9

a: Newcastle-Ottawa scale, BMI: body mass index, WC: waist circumference, WHR: waist-hip ratio, and BP: blood pressure.

**Table 3:** Summary of the influence of different oral contraceptive combination on C-reactive protein in polycystic ovary syndrome^∗^.

Inflammatory marker					
Study	Formulation	Before	After	ES^∗∗^	p^∗∗∗^
CRP (mg/l)					
Morin-Papunen et al, 2003	EE 35 *μ*g/ CPA 2 mg	2.91	4.58	+1.67	<0.001
Chen et al, 2010	EE 35 *μ*g/ CPA 2 mg	0.7	1.2	+0.5	0.002
Teede et al, 2010	EE 35 *μ*g/ CPA 2 mg	3.5	4.7	+1.2	0.001
Ibanez et al, 2011	EE 35 *μ*g/ CPA 2 mg	0.9	1.7	+0.8	<0.05
Gode et al, 2011	EE 35 *μ*g/ CPA 2 mg	3.31	3.87	+0.56	>0.05
Diaz et al, 2012	EE 35 *μ*g/ CPA 2 mg	0.9	2.6	+1.7	<0.05
Ibanez et al, 2013	EE 35 *μ*g/ CPA 2 mg	0.9	3.2	+2.3	<0.01
Christakou et al, 2014	EE 35 *μ*g/ CPA 2 mg	1.36	2.63	+1.27	<0.001
Kahraman et al, 2014	EE 35 *μ*g/ CPA 2 mg	1.21	3.31	+2.1	<0.05
Dardzinska et al, 214	EE 35 *μ*g/ CPA 2 mg	0.77	1.70	+0.93	<0.001
Orio et al, 2016	EE 35 *μ*g/ CPA 2 mg	1.80	1.90	+0.1	>0.05
Tfayli et al, 2011	EE 30 *μ*g/ DRSP 3 mg	1.7	3.8	+2.1	<0.001
Harmanci et al, 2013	EE 30 *μ*g/ DRSP 3 mg	0.50	1.5	+1	<0.05
Kahraman et al, 2014	EE 30 *μ*g/ DRSP 3 mg	0.93	1.22	+0.29	0.040
Christakou et al, 2014	EE 30 *μ*g/ DRSP 3 mg	1.09	1.93	+0.84	<0.001
Yildzhan et al, 2015	EE 30 *μ*g/ DRSP 3 mg	3.77	4.32	+0.55	0.005
Orio et al, 2016	EE 30 *μ*g/ DRSP 3 mg	1.8	1.9	+0.1	<0.05
Vieira et al, 2012	EE 30 *μ*g/ CMA 2 mg	2.1	6.0	+3.9	<0.05
Yildzhan et al, 2015	EE 30 *μ*g/ CMA 2 mg	4.26	0.72	-3.54	0.004
Hoeger et al, 2008	EE 30 *μ*g/ DSG 0.15 mg	6.8	9.5	+2.7	<0.05
Kilic et al, 2011	EE 30 *μ*g/ DSG 0.15 mg	1.67	3.23	+1.56	<0.001
Banaszewska et al, 2007	EE 20 *μ*g/ DSG 0.15 mg	1.61	1.70	+0.09	0.74
Essah et al, 2011	EE 30 *μ*g/ DSG 0.15-0.25 mg	4.9	5.6	+0.7	0.280

^*∗*^All abbreviations are given in the text.

^∗∗^ES: effect-size.

^∗∗∗^p: values taken from each study.

**Table 4:** Influence of different oral contraceptive combination on markers of chronic inflammation in polycystic ovary syndrome^∗^.

Inflammatory marker					
Study	Formulation	Before	After	ES^∗∗^	p^∗∗∗^
IL-6 (pg/ml)					
Diaz et al, 2012	EE 35 *μ*g/ CPA 2 mg	1.67	1.39	-0.28	0.911
Ibanez and de Zegher, 2004	EE 30 *μ*g/ DRSP 3 mg	0.95	1.09	+0.14	>0.05
Vieira et al, 2012	EE 30 *μ*g/ CMA 2 mg	1.90	1.70	-0.20	>0.05
Glintborg et al, 2014	EE 30 *μ*g/ DSG 0.15 mg	1.70	1.40	-0.30	>0.05
Essah et al, 2011	EE 30 *μ*g/ NGM 0.15-0.25 mg	2.10	1.60	-0.50	0.060
PAI-1 (mg/ml)/(IU/ml)					
Hoeger et al, 2008	EE 30 *μ*g/ DSG 0.15 mg	46.9	29.5	-17.4	<0.05
Essah et al, 2011	EE 30 *μ*g/ NGM 0.18-25 mg	81.2	76.5	-04.7	0.520
Orio et al, 2016	EE 30 *μ*g/ DRSP 3 mg	02.6	02.4	-0.2	>0.05
Neutrophils (nx1000/mm^3^)					
Ibanez et al, 2005	EE 30 *μ*g/ DRSP 3 mg/ GSD 75 *μ*g	03.9	04.7	+0.80	>0.05
Lymphocytes (nx1000/mm^3^)					
Ibanez et al, 2005	EE 30 *μ*g/ DRSP 3 mg/ GSD 75 *μ*g	02.3	02.2	-0.1	>0.05
TNF-*α* (pg/ml)					
Vieira et al, 2012	EE 30 *μ*g/ CMA 2 mg	10.6	12.0	+1.4	>0.05
MCP-1 (pg/ml)					
Glintborg et al, 2014	EE 30 *μ*g/ DSG 0.15 mg	50.0	44.0	-06.0	>0.05
sICAM-1(ng/ml)					
Bilgir et al, 2009	EE 35 *μ*g/ CPA 2 mg	370.1	364.7	-5.4	0.800
sVCAM-1(ng/ml)					
Bilgir et al, 2009	EE 30 *μ*g/ DSG 0.15 mg	583.0	522.0	-61.0	0.003
Banaszewska et al, 2007	EE 30 *μ*g/ CMA 2 mg	776.3	742.9	-33.4	0.100
Se-selectin (ng/ml)					
Bilgir et al, 2009	EE 35 *μ*g/ CPA 2 mg	29.1	37.1	+8.0	0.100
Sp-selectin (ng/ml)					
Bilgir et al, 2009	EE 35 *μ*g/ CPA 2 mg	229.4	189.6	-39.8	0.080
sCD40L (ng/ml)					
Kebapcilar et al, 2010	EE 35 *μ*g/ CPA 2 mg	1.33	2.70	+1.37	0.011

^*∗*^All abbreviations are given in the text.

^∗∗^ES: effect-size.

^∗∗∗^p: values taken from each study.
